# Nomenclature for radiopharmaceuticals, consultation of your opinion!

**DOI:** 10.1186/s41181-017-0022-z

**Published:** 2017-01-18

**Authors:** Philip H. Elsinga

**Affiliations:** University of Groningen, Groningen, Netherlands

For many years when attending radiopharmaceutical chemistry-related meetings and reviewing abstracts and papers, I have not always found it easy to reconstruct what the authors exactly mean in their results sections. Are the reported radiochemical yields decay corrected or not? If so to what timepoint? A number of papers have used terms ‘radiochemical conversion’. However, different groups seem to define this in slightly different ways, and others not at all. Furthermore, how should we use superscripts for radionuclide descriptors? Square brackets, no brackets, hyphens? And the list goes on ……

Recognising the increasing challenge in trying to understand radiochemistry communications, the Drug Development Committee of the EANM composed a working group together with representatives of many relevant societies including EANM, SNMMI, SRS and national nuclear medicine societies 2 years ago with the aim to generate consensus on how to use scientific radiochemistry terms/nomenclature and when (or not) to use them. The working group has now published a consultation document on radiochemistry nomenclature good practice and are requesting the radiopharmaceutical chemistry community to review this and feedback by 31st January 2017 in order to finalise the consensus nomenclature document.

I would like to draw your attention to this important initiative and encourage you to read the documents and provide feedback. This initiative will help to enable unambiguous communication in our field. The consultation document and call for feedback can be found on the following link: http://www.srsweb.org/nomenclature-guidelines/

Editor-in-Chief, Philip Elsinga

